# Population activity structure of excitatory and inhibitory neurons

**DOI:** 10.1371/journal.pone.0181773

**Published:** 2017-08-17

**Authors:** Sean R. Bittner, Ryan C. Williamson, Adam C. Snyder, Ashok Litwin-Kumar, Brent Doiron, Steven M. Chase, Matthew A. Smith, Byron M. Yu

**Affiliations:** 1 Department of Electrical and Computer Engineering, Carnegie Mellon University, Pittsburgh, Pennsylvania, United States of America; 2 Department of Biomedical Engineering, Carnegie Mellon University, Pittsburgh, Pennsylvania, United States of America; 3 Center for the Neural Basis of Cognition, Carnegie Mellon University, Pittsburgh, Pennsylvania, United States of America; 4 Department of Bioengineering, University of Pittsburgh, Pittsburgh, Pennsylvania, United States of America; 5 School of Medicine, University of Pittsburgh, Pittsburgh, Pennsylvania, United States of America; 6 Department of Mathematics, University of Pittsburgh, Pittsburgh, Pennsylvania, United States of America; 7 Department of Ophthamology, University of Pittsburgh, Pittsburgh, Pennsylvania, United States of America; 8 Center for Theoretical Neuroscience, Columbia University, New York, New York, United States of America; Northwestern University, UNITED STATES

## Abstract

Many studies use population analysis approaches, such as dimensionality reduction, to characterize the activity of large groups of neurons. To date, these methods have treated each neuron equally, without taking into account whether neurons are excitatory or inhibitory. We studied population activity structure as a function of neuron type by applying factor analysis to spontaneous activity from spiking networks with balanced excitation and inhibition. Throughout the study, we characterized population activity structure by measuring its dimensionality and the percentage of overall activity variance that is shared among neurons. First, by sampling only excitatory or only inhibitory neurons, we found that the activity structures of these two populations in balanced networks are measurably different. We also found that the population activity structure is dependent on the ratio of excitatory to inhibitory neurons sampled. Finally we classified neurons from extracellular recordings in the primary visual cortex of anesthetized macaques as putative excitatory or inhibitory using waveform classification, and found similarities with the neuron type-specific population activity structure of a balanced network with excitatory clustering. These results imply that knowledge of neuron type is important, and allows for stronger statistical tests, when interpreting population activity structure.

## Introduction

Excitatory and inhibitory neurons appear to perform distinct roles in cortical networks. Excitatory neurons form long range synaptic projections and have clustered connectivity with other excitatory neurons [1–4]; inhibitory neurons form local, dense, and non-specific connections [5–7]. Excitatory neurons are almost exclusively pyramidal cells, while inhibitory neurons form a diverse class with multiple subtypes [8–10]. In many cases, inhibitory neurons exhibit greater firing rates than excitatory neurons [11, 12], more attentional modulation [13, 14], and a lesser degree of burstiness [15, 16]. Excitatory neurons are thought to be critical for long-term synaptic potentiation [17], whereas inhibitory neurons are instead postulated to modulate stimulus response gain, sharpen tuning to stimuli, and pace cortical oscillations [18]. Despite these anatomical, statistical, and functional differences, the network-level roles of excitation and inhibition and their interaction are incompletely understood.

In this study, we sought to understand how the patterns of activity produced by a population of neurons (referred to as *population activity structure*) depend on neuron type. Such insights would help to bridge the gap between single-neuron (and pairwise) response properties of different neuron types and their network-level interactions. We leveraged spiking network models, where the type of each neuron and the network connectivity is known, to address this question. We considered two types of balanced networks: one with uniform connectivity (“non-clustered network”) and one with clustered connectivity among excitatory neurons (“clustered network”). This allows us to assess how our results depend on the underlying architecture. Classic non-clustered networks balance excitatory and inhibitory input currents and have been widely studied. The model neurons exhibit Poisson-like spiking variability and zero-mean spike count correlations [19–21]. Clustered balanced networks reproduce additional properties of the spiking variability of biological neurons and have more realistic anatomical structure [22–24]. In this study, we analyzed one representative network of each type, and refer to them as the *clustered network* and the *non-clustered network*.

One way to characterize population activity structure is through the use of dimensionality reduction [25]. Dimensionality reduction methods have been utilized to examine neural population activity during motor control [26, 27], decision-making [28, 29], visual attention [30], and other behavioral tasks [31–35]. These methods characterize the multi-dimensional patterns of activity produced by a population of neurons, which can then be related to stimulus or behavior. For this study, we used factor analysis (FA), which is well-suited for analyzing spiking variability because it separates spiking variability into a component that is shared among neurons and one that is independent across neurons [36, 37]. Shared variability is of particular importance because it is the most likely to be transmitted to downstream neurons and affect neuronal coding [38]. We used FA to measure two characteristics of population activity: *shared dimensionality* and *percent shared variance*. Shared dimensionality measures the number of dimensions in which the shared activity resides. Large values of shared dimensionality indicate a richness to the interactions among neurons, while values of zero indicate approximate independence. Percent shared variance assesses the degree to which the shared activity explains the total variability in the population.

We started by characterizing the activity structure of excitatory and inhibitory populations in the balanced networks by applying FA to samplings of only-excitatory and only-inhibitory neurons while varying the number of neurons and trials sampled. This extends our recent study of only excitatory neurons in model networks [39], and allowed us to compare the multi-dimensional activity structure of the two neuron types in both networks. Then, using the same two networks, we considered samplings of mixed neuron type by varying the ratio of excitatory to inhibitory neurons sampled from each network. By applying factor analysis to the population activity, we observed measurably different population activity structure for various neuron type samplings. In order to ground these model network results with real data, we applied the same analysis to the activity of a population of neurons recorded from the primary visual cortex. By classifying neurons based on waveform shape [14], we found that the broad-spiking and narrow-spiking neurons have similar activity structure as the excitatory and inhibitory populations, respectively, in the clustered network. These results suggest that the identification of neuron types in experimental data can provide a more nuanced understanding of population activity structure.

## Results

**Fig 1:**
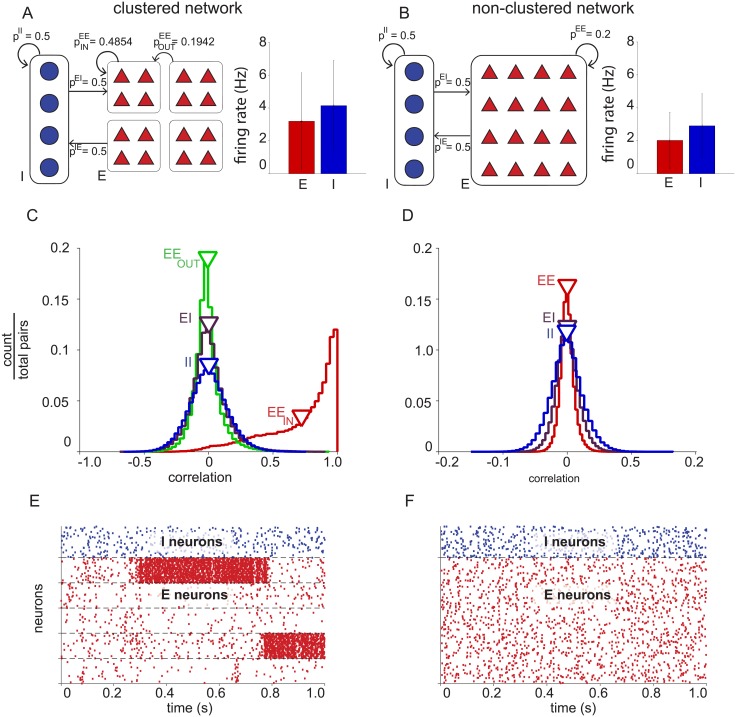
Basic properties of excitatory and inhibitory neurons in balanced networks. (A) Clustered network connectivity (left) of inhibitory-inhibitory (*p^II^*), excitatory-to-inhibitory (*p^IE^*), inhibitory-to-excitatory (*p^EI^*), same-cluster excitatory-excitatory ($p_{IN}^{EE}$), and out-of-cluster excitatory-excitatory ($p_{OUT}^{EE}$) neuron pairs. Average firing rates (right) of excitatory (red, 3.2 ± 2.9 Hz, mean ± standard deviation) and inhibitory (blue, 4.1 ± 2.7 Hz) neurons. (B) Non-clustered network connectivity (left) and average firing rates (right) of excitatory (red, 2.0 ± 1.7 Hz) and inhibitory (blue, 2.9 ± 1.9 Hz) neurons. (C) Clustered network spike count correlations (one second time bins) between same-cluster excitatory-excitatory pairs (*EE_in_*, red, *r* = 0.72 ± 0.27, mean ± standard deviation), out-of-cluster excitatory-excitatory pairs (*EE_out_*, green, *r* = -0.0085 ± 0.11), excitatory-inhibitory pairs (*EI*, purple, *r* = 9.0^−4^ ± 0.14), and inhibitory-inhibitory pairs (*II*, blue, *r* = 4.5^−4^ ± 0.15). (D) Non-clustered network spike count correlations (one second spike bins) between excitatory-excitatory pairs (*EE*, red, *r* = 1.9^−5^ ± 0.11), excitatory-inhibitory pairs (*EI*, purple, *r* = 2.1^−4^ ± 0.018), and inhibitory-inhibitory pairs (*II*, blue, *r* = -7.3^−4^ ± 0.025). Note that the horizontal axis differs between panels C and D. (E) Clustered network spiking activity. A representative sample of 500 neurons (100 inhibitory neurons and 400 excitatory neurons ordered by cluster membership. (F) Non-clustered network spiking activity. A representative sample of 500 neurons (100 inhibitory and 400 excitatory).

To study the organization of neural responses in populations of excitatory and inhibitory neurons, we examined the properties of the spontaneous activity of model excitatory and inhibitory neurons in balanced networks. Two types of balanced networks were analyzed in this study: one with clustering among excitatory neurons (Fig 1A, “clustered” network) and one with uniform connectivity (Fig 1B, “non-clustered” network). Each balanced network contained 4,000 excitatory and 1,000 inhibitory neurons. In the clustered network, the excitatory neurons were partitioned into 50 non-overlapping clusters of 80 neurons, where same-cluster neurons had a higher probability of connection than out-of-cluster excitatory neurons. In the non-clustered network, excitatory neurons connected with uniform probability to other excitatory neurons. Connectivity within the inhibitory population, as well as connectivity between the excitatory and inhibitory populations was uniform in each network.

Both networks exhibit features similar to physiological recordings. The average firing rate of inhibitory neurons was greater than that of excitatory neurons in both model networks (Fig 1A and 1B, right) [11, 12]. Same-cluster excitatory neurons in the clustered network recurrently excite each other, acting similarly to a bistable unit with high and low activity states (Fig 1E) resulting in positive noise correlations which have been observed among nearby neurons [40]. The non-clustered network does not have this property (Fig 1F).

### Excitatory and inhibitory populations of balanced networks have different activity structure

**Fig 2:**
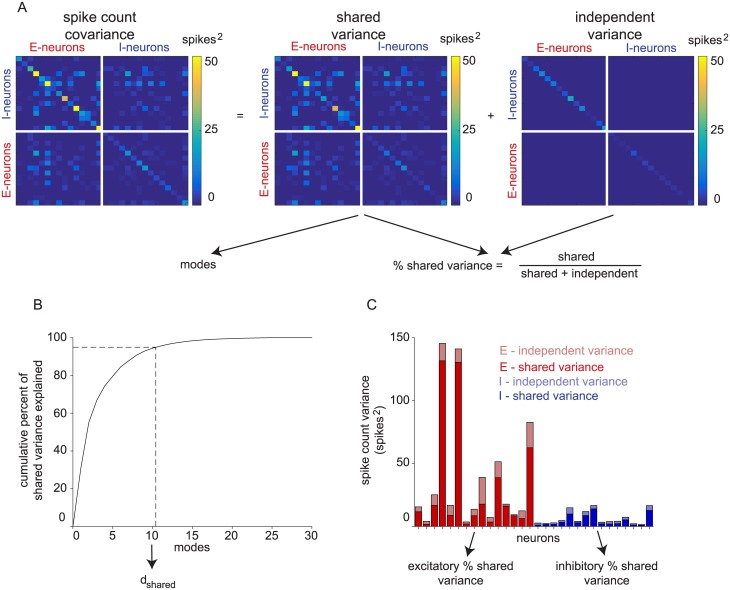
Calculation of dimensionality and percent shared variance. (A) Factor analysis partitions the spike count covariance of sampled excitatory and inhibitory neurons together into shared and independent components. (B) Shared dimensionality (*d*_*shared*_) is the minimum number of eigenvectors of the shared variance matrix necessary to explain 95% of shared variance. Modes are sorted by shared variance explained along the x-axis. (C) Percent shared variance is the ratio of shared to total variance (i.e., shared / (shared + independent)). The percent shared variance is first computed for each neuron, then averaged across all neurons of the same type.

To understand how neuron type impacts population-level metrics of shared variability, we applied FA to spike counts taken in a one second window of spontaneous activity (referred to as a “trial”) from each network. FA decomposes the spike count covariance of neurons into a shared and independent component (Fig 2A), which enables the computation of shared dimensionality (*d*_*shared*_) and percent shared variance (see Methods). Shared dimensionality is the number of modes of shared co-fluctuations of the population activity (Fig 2B). It is a measure of the complexity of these shared co-fluctuations. Percent shared variance measures how much of each neuron’s spike count variability is shared with at least one other sampled neuron (Fig 2C). By investigating how these metrics depend on the number of neurons and trials sampled, we characterized the scaling properties of the excitatory and inhibitory population activity structure. Note that shared dimensionality and percent shared variance need not go up and down together [39].

We focused here on a spike count window of one second, consistent with many previous studies of spike count correlation [13, 40–43]. To further justify this choice, we found that neurons in both model networks and the *in vivo* recordings exhibited non-zero autocorrelation at lags throughout the range of zero to one second (S1 Fig). In addition, we replicated our analyses with a 100 ms window and found the same trends as with a one second window (see details below).

**Fig 3:**
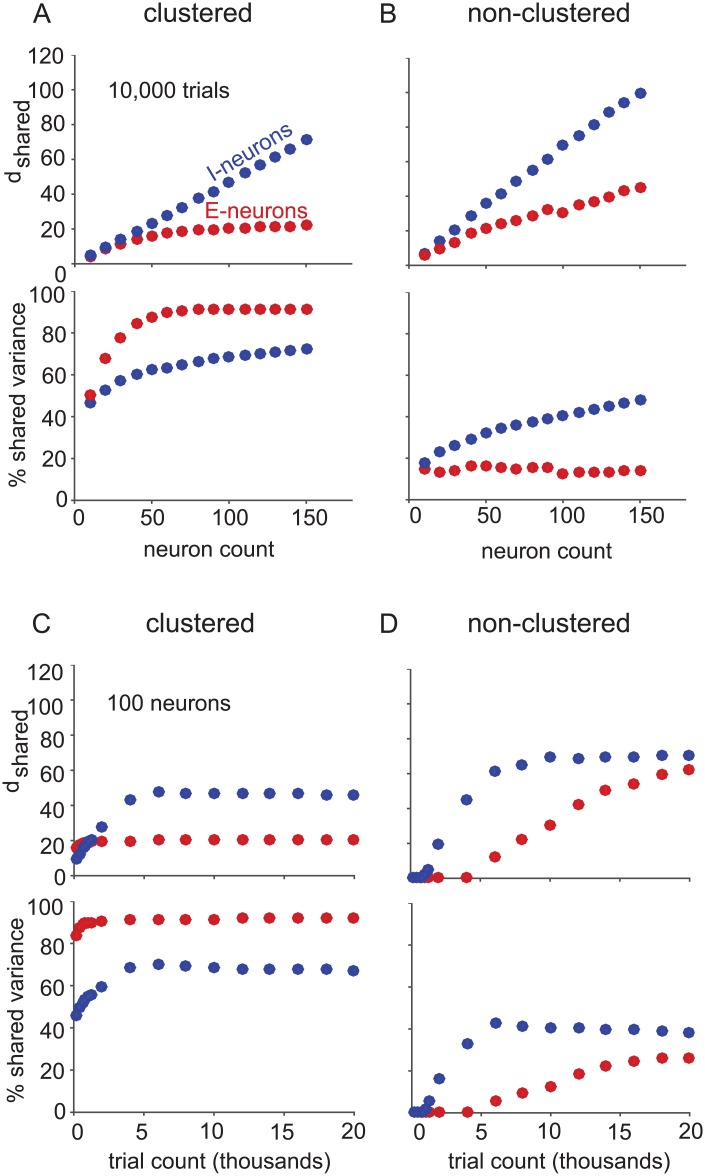
Excitatory and inhibitory population activity structure. (A) Clustered and (B) non-clustered network shared dimensionality (top panels) and percent shared variance (bottom panels) with increasing neuron count with 10,000 trials. Dots (red: excitatory neurons, blue: inhibitory neurons) indicate the mean across five non-overlapping sets of sampled neurons and five non-overlapping sets of trials (25 sets total), and standard error bars are smaller than the dot size in all cases. To assess shared dimensionality and percent shared variance for a larger number of neurons, we grouped the five non-overlapping sets of 150 neurons from each neuron type population of each network into 750-neuron samplings. For 750-neuron samplings of the clustered network (10,000 trials), the excitatory population had a shared dimensionality of 23.95 ± 0.05 and percent shared variance of 91.43% ± 0.06%, while the inhibitory population had a shared dimensionality of 246.7 ± 2.5 and a percent shared variance of 79.56% ± 0.22%. For 750-neuron samplings of the non-clustered network (10,000 trials), the excitatory population had a shared dimensionality of 139.4 ± 3.8 and percent shared variance of 20.70% ± 0.43%, while the inhibitory population had a shared dimensionality of 347.2 ± 4.2 and a percent shared variance of 59.74% ± 0.47%. (C-D) same conventions as A and B, but for increasing trial count with 100 neurons.

The excitatory and inhibitory populations had notable differences in how shared dimensionality scaled with increasing neuron count in both networks. We began by applying FA to only-excitatory (red) and only-inhibitory (blue) neuron samplings of the clustered (Fig 3A) and non-clustered (Fig 3B) networks. The shared dimensionality of the excitatory population saturated with increasing neuron count in the clustered network (Fig 3A, top, red), but not in the non-clustered network (Fig 3B, top, red) [39]. In contrast, the shared dimensionality of the inhibitory population continued to increase with neuron count regardless of whether the excitatory population was clustered or not (Fig 3A and 3B, top, blue). These scaling properties are related to the connectivity of the neurons. For those populations with uniform connectivity (inhibitory in both networks and excitatory in the non-clustered network), more dimensions are revealed as more neurons are sampled. In contrast, the shared activity among the excitatory neurons in the clustered network is dominated by the between-cluster interaction. We previously showed that, although there are 50 modes describing the interaction among the 50 clusters, the top 20 modes explain 95% of the shared variance [39]. Thus, the asymptotic number of shared dimensions (20) is smaller than the number of clusters (50).

To further characterize the excitatory and inhibitory population activity structure, we sought to measure the prominence of the shared co-fluctuations described by the identified dimensions. We measured the percentage of each neuron’s spike count variance that was explained by the shared dimensions. Note that, for a given shared dimensionality, the percent shared variance can be large or small depending on how strongly those dimensions modulate each neuron’s activity. We found that the excitatory population had greater percent shared variance than the inhibitory population in the clustered network (Fig 3A, bottom). This is reasonable because the clustering of the excitatory population leads to same-cluster neurons increasing and decreasing their activity together (cf. Fig 1C), which would tend to increase the amount of one neuron’s variability that can be explained by other neurons in the same cluster. Conversely, in the non-clustered network, the inhibitory population had greater percent shared variance than the excitatory population (Fig 3B, bottom). This observation is related to the greater variance of the spike count correlation distribution for inhibitory-inhibitory than excitatory-excitatory neuron pairs (cf. Fig 1D). We suspected that these observations were connected, since increasing the variance of a zero-mean spike count correlation distribution implies an increase in the number of neuron pairs with non-zero spike count correlation, which consequently increases percent shared variance. To demonstrate this, we generated population spike counts corresponding to spike count correlation distributions of different variances using the method described in [44]. We found that percent shared variance indeed increases with the variance of the spike count correlation distribution (S2 Fig). Furthermore, we observed that the percent shared variance of the inhibitory neurons increased with neuron count for both networks (Fig 3A and 3B, bottom, blue), in contrast to that of excitatory neurons which saturated with increasing neuron count (Fig 3A and 3B, bottom, red) [39]. In other words, more of the spike count variability among inhibitory neurons was explained as more inhibitory neurons were sampled.

Having examined how shared dimensionality and percent shared variance changed with neuron count for excitatory versus inhibitory population samplings, we next examined how these two quantities changed with trial count, while keeping the number of neurons fixed. This can help us understand how much data is required to fully identify the shared activity of the sampled neurons. For both neuron types in both networks, the shared dimensionality and percent shared variance saturated with increasing trials sampled. The number of trials at which shared dimensionality or percent shared variance saturated was related to how “salient” the shared population activity structure was in the raw spike counts. When the shared population activity structure was more salient, fewer trials were needed to identify the shared structure. In the clustered network, the shared dimensionality and percent shared variance of the excitatory population (Fig 3C, red) saturated at fewer trials than the inhibitory population (Fig 3C, blue). This is related to the fact that the excitatory population had greater percent shared variance, and therefore more salient shared activity structure, than the inhibitory population. In the non-clustered network, we saw the opposite trend: inhibitory population samplings (Fig 3D, blue) saturated in dimensionality and percent shared variance with fewer trials than the excitatory population samplings (Fig 3D, red). In the non-clustered network, the inhibitory population had more salient shared activity structure than the excitatory population, as indicated by the higher percent shared variance of the inhibitory population. The asymptotic dimensionalities in Fig 3C and 3D depend on the number of neurons analyzed (in this case, 100 neurons). If more neurons are included, the asymptotic dimensionality would be higher for inhibitory neurons in the clustered network and both types of neurons in the non-clustered network, as indicated by Fig 3A and 3B.

Because the activity of same-cluster excitatory neurons tends to increase and decrease together, we expected excitatory neurons to have greater percent shared variance in the clustered than in the non-clustered network (compare red curves in Fig 3C and 3D, bottom). Interestingly, we found that this was also true for the inhibitory neurons (compare blue curves in Fig 3C and 3D, bottom), even though the probability of connections involving inhibitory neurons was the same in the two networks. Thus clustering structure among excitatory neurons resulted in shared variability that propagated to the inhibitory population, resulting in increased shared variability in the inhibitory population. This is consistent with the greater variance of the distribution of spike count correlations between inhibitory-inhibitory neuron pairs in the clustered network (cf. Fig 1C, blue) than those in the non-clustered network (cf. Fig 1D, blue). All the trends observed in Fig 3 with one second spike count windows remained true with 100 ms spike count windows (S3 Fig).

### Excitatory clustering affects modes of shared activity in both the excitatory and inhibitory populations

**Fig 4:**
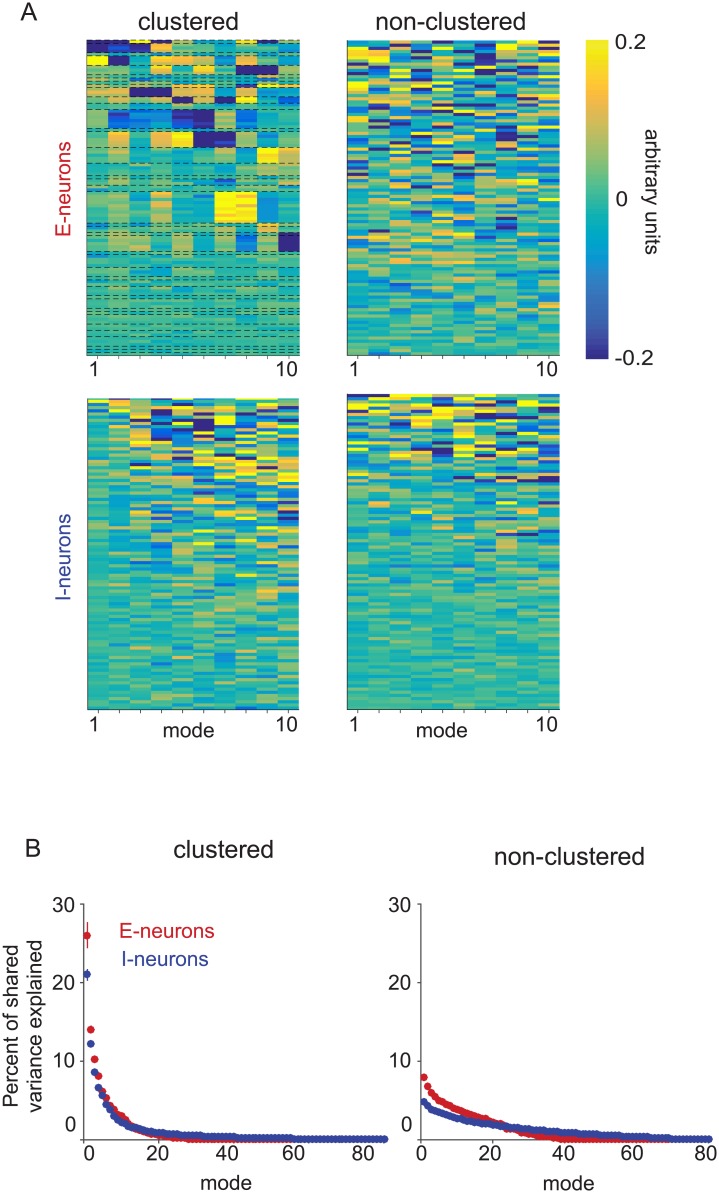
Modes of shared activity. (A) Modes of only-excitatory (top) and only-inhibitory (bottom) 100-neuron samplings from the clustered (left) and non-clustered (right) networks. Columns of each heatmap represent the eigenvectors of the shared covariance matrix, ordered by the amount of shared variance explained. Each eigenvector is a unit vector, and so its entries have arbitrary units. Each row corresponds to a neuron, and neurons are ordered from highest to lowest mean firing rate. For excitatory neurons from the clustered network (top left), neurons were additionally grouped by cluster. Some clusters were represented by more neurons than other clusters due to random sampling. (B) Percent of shared variance explained by each mode for 100-neuron analyses of the excitatory (red) and inhibitory (blue) populations in the clustered (left) and non-clustered (right) networks with 10,000 trials. Dots indicate the mean across five non-overlapping sets of neurons and five non-overlapping sets of trials (25 sets total) and error bars indicate standard error (not visible for most data points).

To further characterize the excitatory and inhibitory population activity structure, we studied the modes (or dimensions) of shared activity of each population in the two networks. For populations of each neuron type from each network architecture, the ten most dominant modes of shared activity (eigenvectors of the shared covariance matrix) are displayed in order of the amount of shared variance explained by each mode. (Fig 4A). The excitatory neurons are ordered by cluster membership (Fig 4A, top-left). Same-cluster neurons had similar values within each mode for the excitatory population of the clustered network, indicating that the dominant modes describe co-varying activity between clusters. In contrast, the excitatory population of the non-clustered network (Fig 4A, top-right) and both inhibitory populations (Fig 4A, bottom) did not exhibit any obvious structure in the modes of shared activity.

Although there were no differences in mean inhibitory connection properties between the clustered and non-clustered networks, we found that excitatory clustering changes the prominence of the dominant modes of shared activity in the inhibitory population. In previous work, we found that dominant modes of shared activity in the excitatory population of the clustered network explained large proportions of shared variance (Fig 4B, left, red), in contrast to those in the excitatory population in the non-clustered network where shared variance was distributed more equally across modes (Fig 4B, right, red) [39]. The five most dominant modes of shared activity explained 64.5% ± 1.5% of shared variance in the clustered network and 31.2% ± 0.8% of shared variance in the non-clustered network. Interestingly, we found that the dominant modes of the inhibitory population in the clustered network also explained a greater percentage of shared variance (Fig 4B, left, blue) than in the non-clustered network (Fig 4B, right, blue). For inhibitory populations, the five most dominant modes of shared activity explained 54.3% ± 0.4% of shared variance in the clustered network and only 20.1% ± 0.2% of shared variance in the non-clustered network. Using FA, we were able to observe that shared activity structure induced by clustering in the excitatory population also propagated to the inhibitory population, which had the same connectivity structure in the two models.

### Ratio of excitatory to inhibitory neurons affects population activity structure of mixed-type samplings

While sampling strictly from populations of a single neuron type is useful for characterizing the role of neuron type in balanced network activity, realistic population samplings likely contain a mixture of both excitatory and inhibitory neurons. In this section, we explore how the population activity structure depends on the ratio of excitatory to inhibitory neurons sampled from the clustered and non-clustered networks. The same networks with 4,000 excitatory and 1,000 inhibitory neurons were used, but we analyzed 100-neuron samplings with different ratios of excitatory to inhibitory neurons.

**Fig 5:**
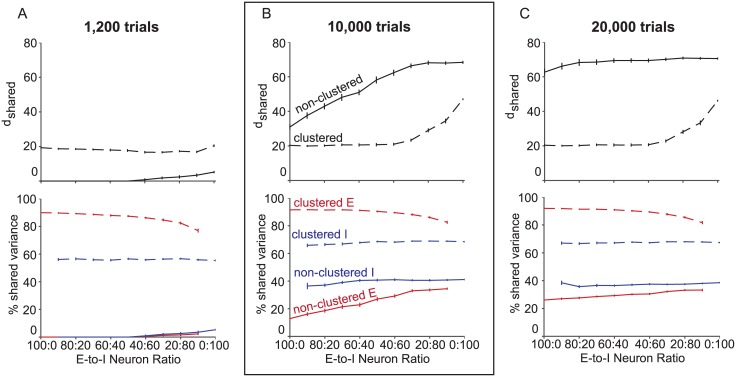
Mixed neuron type samplings. Population activity structure is dependent on the ratio of excitatory to inhibitory neurons sampled. *d*_*shared*_ (top panels) and percent shared variance (bottom panels) averaged across excitatory neurons (red) and inhibitory neurons (blue) for different ratios of excitatory to inhibitory neurons sampled from the clustered (dashed) and non-clustered (solid) networks for 100 neurons and (A) 1,200, (B) 10,000, and (C) 20,000 trials sampled. The left and right ends of the horizontal axes indicate only-excitatory and only-inhibitory samplings, respectively. Panel B is boxed because shared dimensionality and percent shared variance for the 100:0 and 0:100 ratios correspond to values shown in Fig 3 (100 neurons, 10,000 trials).

The ratio of excitatory to inhibitory neurons affected the shared dimensionality of population samplings. As we replaced excitatory neurons with inhibitory neurons (Fig 5, moving left to right along the horizontal axis), shared dimensionality increased from the only-excitatory neuron sampling to the only-inhibitory neuron sampling for the clustered and non-clustered networks with 10,000 trials (Fig 5B, top). The endpoints of these curves are the shared dimensionalities of the purely excitatory (cf. Fig 3A and 3B, 100 neurons, top, red) and purely inhibitory (cf. Fig 3A and 3B, 100 neurons, top, blue) populations considered before.

The ratio of excitatory to inhibitory neurons sampled affected the percent shared variance of excitatory (red) and inhibitory (blue) neurons in the clustered (dashed) and non-clustered (solid) networks with 10,000 trials (Fig 5B, bottom). Since percent shared variance measures how much of a neuron’s activity covaries with at least one other sampled neuron, it is possible for the percent shared variance of excitatory and inhibitory neurons to change based on how many of each type of neuron is also sampled. Consider the percent shared variance of excitatory neurons when going from only-excitatory samplings to only-inhibitory samplings (left to right along the horizontal axis). As excitatory neurons are replaced by inhibitory neurons, the percent shared variance of the remaining excitatory neurons would decrease if they are more highly correlated with excitatory neurons being replaced in the sampling than the newly sampled inhibitory neurons. Conversely, the percent shared variance of the remaining excitatory neurons would increase if they are more highly correlated with the newly sampled inhibitory neurons than the replaced excitatory neurons. In the clustered network, we found that as we replaced excitatory neurons with inhibitory neurons, the percent shared variance for excitatory neurons decreased (Fig 5B, bottom, dashed red). This indicated that excitatory neurons in the clustered network shared more of their activity with other excitatory neurons. In contrast, the percent shared variance of excitatory neurons in the non-clustered network (Fig 5B, bottom, solid red) increased as more inhibitory neurons were sampled. This showed that in the non-clustered network, excitatory neurons shared more of their activity with inhibitory neurons than other excitatory neurons. The percent shared variance of inhibitory neurons in both networks was mostly independent of neuron type sampling ratio (Fig 5B, bottom, blue), showing that inhibitory neurons shared their spike count variance equally with excitatory and inhibitory neurons. One might have expected the percent shared variance of inhibitory neurons in the non-clustered network to have greater percent shared variance with other inhibitory neurons than with excitatory neurons, because the distribution of spike count correlations had greater variance for inhibitory-inhibitory pairs (cf. Fig 1D, blue) than for excitatory-inhibitory pairs (cf. Fig 1D, purple). However, the excitatory and inhibitory populations had different distributions of spike count variance, so the relationship between the variance of the spike count correlation distribution and the percent shared variance was more complicated when sampling from both populations instead of one.

We then investigated how the results in (Fig 5B) depend on the number of trials sampled by performing the same analysis with 1,200 (Fig 5A) and 20,000 (Fig 5C) trials. In the non-clustered network, since shared dimensionality and percent shared variance of the excitatory population did not saturate by 10,000 trials (cf. Fig 3D, red), we expected higher and lower trial counts to exhibit different trends of shared dimensionality and percent shared variance with neuron type sampling ratio. With 1,200 trials, the non-clustered network shared dimensionality (Fig 5A, top, solid), as well as the percent shared variance of excitatory (Fig 5A, bottom, solid red) and inhibitory (Fig 5A, bottom, solid blue) neurons, were small for all ratios. With 20,000 trials, the non-clustered dimensionality was flatter than with 10,000 trials, since the shared dimensionality of excitatory neuron dominated ratios had increased nearly to the same level as the inhibitory dominated ratios (Fig 5C, solid). This is consistent with Fig 3D (top), where the excitatory and inhibitory shared dimensionalities were similar for high trial counts. As with 10,000 trials, with 20,000 trials, excitatory neurons shared more of their spike count variance with inhibitory neurons than other excitatory neurons (Fig 5C, bottom, solid red), and inhibitory neurons shared their spike count variance equally with each neuron type (Fig 5C, bottom, solid blue).

In the clustered network, the trends of shared dimensionality and percent shared variance with neuron type sampling ratio did not change from 10,000 to 20,000 trials (compare Fig 5B, dashed with Fig 5C, dashed), since shared dimensionality and percent shared variance saturated by 10,000 trials for the excitatory and inhibitory populations (cf. Fig 3C). However, with only 1,200 trials instead of 10,000 trials, the shared dimensionality for inhibitory neuron dominated ratios dropped (Fig 5A, top, dashed), and percent shared variance of excitatory and inhibitory neurons slightly decreased (Fig 5A, bottom, dashed), consistent with Fig 3C. With 20,000 trials, as with 10,000 trials, excitatory neurons shared more of their spike count variance with other excitatory neurons than inhibitory neurons (Fig 5C, bottom, dashed red), and inhibitory neurons shared their spike count variance equally between both neuron types (Fig 5C, bottom, dashed blue). Using a spike count window size of 100 ms (S4 Fig), we observed the same trends as in Fig 5.

### Neuron-type information reduces uncertainty of population activity metrics

In practice, we are often blind to the types of the neurons we sample, so we can expect variability in population activity metrics to arise from the variability in the sampled ratio of neuron type. Unknown sampling ratios of neuron type can confound comparisons of population activity structure between two datasets. Consider the conclusion from Fig 5B (top) that the shared dimensionality of the non-clustered network is greater than that of the clustered network. As an extreme example to illustrate the concept, let’s say that only excitatory neurons are sampled from the non-clustered network and only inhibitory neurons are sampled from the clustered network. We would conclude that the clustered network has higher dimensionality than the non-clustered network. Therefore, it is important to know the excitatory versus inhibitory composition of the sampled population to appropriately interpret the measured shared dimensionality.

**Fig 6:**
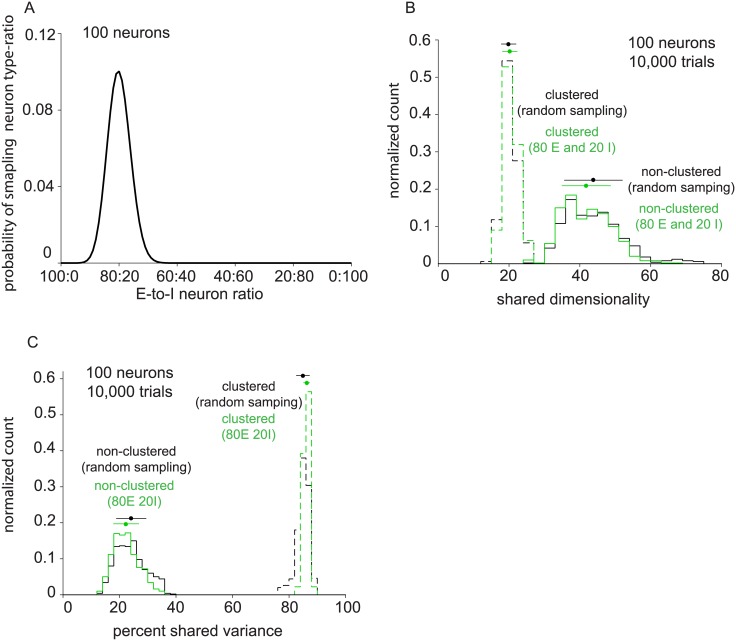
Population activity metrics for samplings that are blind to neuron type. (A) Probability density of neuron type sampling ratio for 100 neuron samplings. (B) Distribution of shared dimensionality of the clustered (dashed black) and non-clustered (solid black) networks for neuron type blind samplings of 100 neurons and 10,000 trials. Distribution of shared dimensionality for 80 excitatory, 20 inhibitory neuron samplings from the non-clustered network (green). (C) Distribution of percent shared variance of the clustered (dashed black) and non-clustered (solid black) networks for neuron type blind samplings of 100 neurons and 10,000 trials.

In less extreme cases, the comparison of shared dimensionality (or percent shared variance) can involve multiple sets of recordings, each of which has a different composition of excitatory and inhibitory neurons. Statistical tests will depend on the variance of the measured shared dimensionality (or percent shared variance) across recordings. If the neuron type composition is unknown, the measurement of shared dimensionality (or percent shared variance) can have high variance, thereby leading to a weak statistical test. However, if the neuron type composition is known, then this can be controlled for in the comparison, leading to a stronger statistical test. To make this concrete, consider randomly sampling 100 neurons from a network with 4,000 excitatory neurons and 1,000 inhibitory neurons, as in the two balanced networks we are working with. The probability of sampling *k* excitatory neurons in an *n*-neuron sample is

$$\begin{array}{c} Pr\left(k|N_{E},N_{I},n\right)=\frac{\left(\begin{array}{c} N_{E}\\ k \end{array}\right)\left(\begin{array}{c} N_{I}\\ n-k \end{array}\right)}{\left(\begin{array}{c} N_{E}+N_{I}\\ n \end{array}\right)} \end{array}$$ (1)

for a network with *N*_*E*_ excitatory neurons and *N*_*I*_ inhibitory neurons. This distribution is depicted in Fig 6A for *N*_*E*_ = 4,000, *N*_*I*_ = 1,000, and *n* = 100. We computed shared dimensionality (Fig 6B) and percent shared variance (Fig 6C) for 100 neuron, 10,000 trial samplings of the clustered and non-clustered networks which were blind to neuron type. The clustered network exhibited shared dimensionality of 20.3 ± 1.8 (Fig 6B, dashed, black, mean ± standard deviation) and percent shared variance of 85.8% ± 1.4% (Fig 6C, dashed, black). The non-clustered network exhibited shared dimensionality of 43.1 ± 8.2 (Fig 6B, solid, black) and percent shared variance of 23.7% ± 5.3% (Fig 6C, solid, black).

Now, suppose that we seek to perform a statistical test involving shared dimensionality for the non-clustered network. If neurons are randomly sampled from the network, the standard deviation is 8.2 (Fig 6B, solid, black). However, if the neuron type composition is known and controlled for (in this case, 80 E and 20 I), the standard deviation decreases to 6.8 (Fig 6B, solid, green). Similarly, the percent shared variance of the clustered and non-clustered networks decreases when neuron type composition is controlled from 2.3 to 1.0 (Fig 6C, dashed) and from 5.2 to 4.5 (Fig 6C, solid), respectively. When sampling neurons randomly from the clustered network, the standard deviation of shared dimensionality is 2.0 (Fig 6B, dashed, black), which is the same as when neuron type ratio is controlled (Fig 6B, dashed, green). This is consistent with the fact that the shared dimensionality of the clustered network is nearly constant (cf. Fig 5B, top, dashed) in the regime of neuron types ratios that are likely to arise from random sampling (Fig 6A). In practice, we cannot usually specify how many excitatory and inhibitory neurons are recorded in an experiment, but we can subsample the neurons after the experiment to obtain the desired neuron type ratio.

### V1 recordings show similar population activity structure as the clustered network

**Fig 7:**
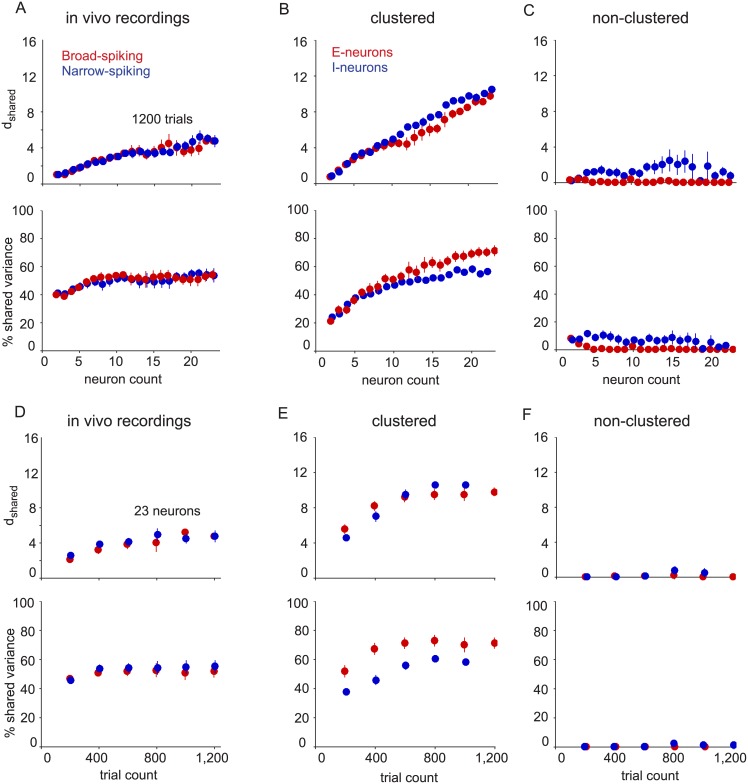
Comparing V1 recordings with network models. (A) V1 recordings. Shared dimensionality (upper panel) and percent shared variance (lower panel) for broad-spiking (red) and narrow-spiking (blue) neurons. Recorded neurons were separated designated as broad-spiking or narrow-spiking using a mixture of Gaussians classifier on average waveform parameters. (B) Same conventions as in (A), but for the clustered network (left panels) and non-clustered network (right panels) based on 1,200 trials for various neuron counts. Red indicates excitatory neurons and blue indicates inhibitory neurons. (C-D) Same conventions as in A-B, but based on 23 neurons and various trial counts. Error bars indicate standard error taken over four arrays for V1 recordings and four sets of non-overlapping samplings for model networks.

We sought to determine whether the differences we observed in model networks were present in the measured activity of excitatory and inhibitory populations *in vivo*. To investigate this, we analyzed spontaneous activity recorded from the primary visual cortex (V1) of anesthetized macaque monkeys [40]. Although the neuron type (excitatory vs. inhibitory) was not known with certainty in these extracellular recordings, previous studies have demonstrated a link between excitatory and inhibitory neuron classes and their extracellular waveform shape [45–47]. This classification approach has revealed a number of functional differences between putative excitatory and inhibitory neurons [13–16, 48]. We therefore adopted this approach (see Methods) to classify broad-spiking neurons (also known as regular-spiking, or putative excitatory) and narrow-spiking neurons (also known as fast-spiking, or putative inhibitory) using a recently published methodology [14]. We analyzed data from arrays implanted in four hemispheres of three animals. Each neuron from these recordings was assigned a probability of being in the broad-spiking or narrow-spiking class based on its average spike waveform (S5 Fig). We identified sets of putative excitatory and inhibitory neurons in these recordings (23 to 47 broad-spiking, 25 to 72 narrow-spiking) by selecting neurons with probability greater than 85% of being in the broad-spiking or narrow-spiking class. In each of the four datasets, there were at least 23 neurons of each type and 1,200 “trials” (i.e., one second windows of spontaneous activity). We fit FA to samplings of only broad-spiking (red) and only narrow-spiking (blue) neurons, which had different average waveforms, for various numbers of neurons and trials sampled (in the same manner as in Fig 3) (Fig 7A and 7D). For comparison, we performed the same analysis on the clustered (Fig 7B and 7E) and non-clustered (Fig 7C and 7F) networks using the same numbers of neurons and trials as in the real data.

The V1 recordings had similar population activity structure to the corresponding populations in the clustered network when compared with 23 neurons of each type and 1,200 trials sampled. The shared dimensionality of the broad-spiking population (red) and narrow-spiking population (blue) increased with increasing neuron count (Fig 7A, top), as did the excitatory (red) and inhibitory (blue) populations of the clustered network (Fig 7B, top). In the non-clustered network, the excitatory and inhibitory populations show no trends in shared dimensionality (Fig 7C, top). The percent shared variance of the broad-spiking and narrow-spiking populations initially increased with neuron count, then plateaued (Fig 7A, bottom). The percent shared variance of both neurons types in the clustered network also increased, and continued to increase in the experimental regime of neuron counts (Fig 7B, bottom). In contrast, the non-clustered network was different from both the V1 recordings and clustered network in that it showed nearly zero shared dimensionality and percent shared variance for both neuron types (Fig 7C).

We also investigated how the activity structure of the narrow-spiking and broad-spiking populations of the V1 recordings varied with trial count. The shared dimensionality of both neuron types in the V1 recordings (Fig 7D, top), as well as the clustered network (Fig 7E, top), increased and eventually plateaued with increasing trial count. Similarly the percent shared variance of both populations plateaued with increasing trial count in both the V1 recordings (Fig 7D, bottom) and the clustered network (Fig 7E, bottom). As in Fig 7C, the non-clustered network showed no trends in shared dimensionality or percent shared variance due to the small number of trials (Fig 7F).

Overall, the trends of the V1 recordings were similar to those of the clustered network. However, there were two notable discrepancies between the V1 recordings and clustered network. First, whereas the excitatory neurons showed higher percent shared variance than inhibitory neurons in the clustered network (Fig 7B and 7E, bottom), this was not seen in the V1 recordings (Fig 7A and 7D, bottom). We speculate that this difference could be due to the connectivity structure of the clustered network, or our ability to classify neurons accurately based on waveform shape (see Discussion). Second, the mode that explained the largest amount of shared variance (referred to as the “dominant mode”) in the V1 recordings described the population increasing and decreasing its activity together (S6 Fig, left column of heatmaps have nearly all elements of the same sign). Previous studies have demonstrated this by analyzing all recorded neurons together [39, 49]; here, we showed that this is also true when analyzing broad-spiking and narrow-spiking populations separately. However, the dominant modes for the excitatory and inhibitory populations in the clustered network (Fig 4A, left) did not exhibit this property.

## Discussion

In this work, we used spiking network models to examine how the patterns of activity produced by a population of neurons depend on neuron type (excitatory and inhibitory). We found that excitatory and inhibitory neurons showed different population activity structure. Notably, the inhibitory population expressed more modes of shared activity than the excitatory population in both networks that we implemented. Furthermore, the population activity structure depended on the ratio of excitatory to inhibitory neurons sampled. To ground the network results with real data, we classified V1 neurons based on waveform and found that the population activity structure of each neuron type in the *in vivo* recordings better resembled that of the clustered network. Overall, these results demonstrate that knowledge of neuron type is important, and allows for stronger statistical tests, when interpreting population activity structure.

Although many spiking network models have been proposed, we considered models where strong excitation is balanced by an equally strong and opposing inhibition. Previous work on balanced networks has focused on the response properties of the excitatory population [20, 21, 39, 50, 51] because, in balanced networks, the inhibitory population simply tracks the excitatory population. The tracking property of the inhibitory population is essential for network stability and yields network activity that is asynchronous [21]. Thus, analyzing inhibition should not, in principle, provide any further insight about network dynamics than the excitatory population. This intuition is developed from the theory of balanced networks with homogeneous connectivity (i.e., non-clustered) where typically spike train statistics are only studied in the limits of large network size and large trial counts [20, 21, 24]. In our study, we compared and contrasted the network spiking statistics of a non-clustered balanced network to one with clustered excitatory connectivity [22]. Counter to classical intuition, analysis of both networks shows significant differences in the activity structure of the excitatory and inhibitory populations.

We focused on the trends in shared dimensionality and percent shared variance to compare the population activity structures of different neuron types. Absolute levels of shared dimensionality and percent shared variance obtained for the model networks are likely to depend on model parameters, such as the number of clusters, the synaptic weights, and the probability of synaptic connection. We used the parameters described in [22], and we did not try to fit these parameters so that the results would match those found from experimental data. Understanding the trade-offs among the different model parameters necessary to reproduce the values of shared dimensionality and percent shared variance measured for the *in vivo* recordings would be an interesting avenue of future research.

In our previous work, we attempted to group the neurons from the V1 recordings into clusters based on the modes of shared activity [39]. However, we did not find clear groups of neurons. In a separate analysis, using the clustered network, we asked how the number of clusters represented in the neuron sample affects the population activity structure [39]. We found that the shared dimensionality increases with the number of clusters represented, but the percent shared variance does not depend on the number of clusters represented.

Recently, several studies have found that networks with heterogenerous connectivity can better reproduce the variability and dynamics of cortical activity [22–24, 52]. The clustered network that we studied here is an example of such a heterogeneous network. While inhibitory projections do not appear to be structured [7], there may be structured projections from excitatory clusters to an inhibitory target [53]. How structured projections from excitatory to inhibitory neurons shape the structure of population activity would be an interesting line of further research. In a similar vein, balanced network models with connectivity that depends upon the spatial distance between neurons have been developed [54] and can serve as an interesting platform for future studies of neural population activity structure. A complete understanding of these differences between balanced networks with homogeneous and heterogeneous wiring will require a full theory of heterogeneous balanced networks [24].

A central goal of network modeling is to construct models which produce activity that resemble *in vivo* recordings. To date, most studies have compared network models with real data at the level of individual neurons (e.g., averaged firing rate across neurons or Fano factor) and pairs of neurons (e.g., pairwise correlation) [20–22, 55]. To move beyond single-neuron and pairwise metrics, we compare here the joint activity patterns across a population of neurons, as in recent studies [23, 39, 52, 56]. This comparison is facilitated by dimensionality reduction methods (in this case, factor analysis), which produce summary statistics (in this case, shared dimensionality and percent shared variance) of the population activity structure. This approach is likely to be beneficial for the design of future network models. In addition to matching single-neuron and pairwise metrics, network models should reproduce the population activity structure of *in vivo* recordings.

The balanced networks studied here are deterministic and chaotic. Although the model neurons do not each have an independent noise source, FA identifies “independent variance” for each neuron’s activity that cannot be predicted from the other sampled neurons using a linear predictor. The independent component of variability arises from the fact that i) only a subset of the neurons in the network are sampled, and ii) the balanced networks have non-linear dynamics that are not captured by a linear and static FA model.

In our V1 recordings, the difference in population activity structure between broad-spiking neurons and narrow-spiking neurons was small (cf. Fig 6). One possibility is that there is no difference in the population activity structure between excitatory and inhibitory neurons in cortical networks. Another possibility (which we think is more likely) is that there is a difference, but we were not able to see it in our data for two reasons. First, we were limited in sampled neurons (23) and trials (1,200). In the clustered network, we observed that there was a substantial difference in population activity structure between excitatory and inhibitory neurons for large numbers of neurons (100) and trials (10,000) (cf. Fig 3). However, when the number of neurons and trials was reduced to that available in V1 recordings (23 neurons and 1,200 trials), the difference between excitatory and inhibitory neurons was much smaller (cf. Fig 6). This is consistent with the trends observed in the V1 recordings. Another possible reason is that neurons might be misclassified based on waveform shape. New technologies are being developed that can identify neuron type more definitively *in vivo* using optogenetics (e.g., [57, 58]) or selectively expressing fluorescent indicators in certain neuron types for optical imaging (e.g., [59, 60]). Given the rapid development and adoption of these technologies, there is an ever-growing need for methods and analyses like those presented here to understand how excitatory and inhibitory populations interact [61].

The properties of excitatory versus inhibitory neurons have largely been studied at the cellular or sub-cellular levels. Applying dimensionality reduction methods to samplings of the same neuron type allows us to link differences at the sub-cellular and cellular levels to population-level effects at the network level. Differences in connectivity patterns, projection lengths, and firing variability may all contribute to differences in population activity structure. Our study connects two disparate, yet related, fields of neuroscience by studying population activity as a function of neuron type sampling.

Classifying neurons as excitatory or inhibitory is only a first-level characterization of neuron type. There exist multiple types of inhibitory neurons which vary in terms of morphological, physiological, and neurochemical characteristics [62]. These different subtypes have been implicated in different roles at the network level [63, 64], and are being incorporated in network models [65]. The current study can be extended to assess whether there are signatures of the different inhibitory neuron types in the population activity structure, and whether it is necessary to sample all types of inhibitory neurons to understand network function. Furthermore, we focused on spontaneous activity for the models networks (i.e., zero inputs) and in vivo recordings (i.e., no visual stimulus). The approach presented here can be used to compare the population activity structure of excitatory and inhibitory neurons for evoked activity, i.e., model networks with non-zero inputs and neural responses to visual stimuli.

## Materials and methods

### Balanced spiking networks

We considered two spiking networks: a classic balanced network (“non-clustered network”) and a network in which the excitatory neurons exhibited clustered structure (“clustered network”). Spiking network activity was generated using the same parameters as in [22]. Both networks contained 4,000 excitatory and 1,000 inhibitory leaky integrate-and-fire neurons. In the clustered network, there were 50 clusters, each with 80 excitatory neurons. The same-cluster excitatory neurons had higher probability of connection ($p_{IN}^{EE}=0.485$) than out-of-cluster excitatory neurons ($p_{OUT}^{EE}=0.194$). In the non-clustered network, all excitatory neurons had a uniform probability of connection (*p*^*EE*^ = 0.2). All other neuron type connection probabilities were uniform for both networks (*p*^*EI*^ = *p*^*IE*^ = *p*^*II*^ = 0.5). The membrane potential *V* of each neuron was modeled by the following differential equation

$$\begin{array}{c} \dot{V}=\frac{1}{\tau }\left(\mu -V\right)+I_{syn} \end{array}$$ (2)

where *μ* is the voltage bias, *I*_*syn*_ is the synaptic input current, and *τ* is a time constant that is dependent on neural type (15 ms for excitatory neurons, 10 ms for inhibitory neurons). When *V* = *V*_*th*_ = 1, the neuron fires, and is set to *V*_*re*_ = 0 for a refractory period of 5 ms. The synaptic input current to a particular neuron *i* from population *x* was modeled via

$$\begin{array}{c} I_{i,syn}^{x}\left(t\right)=\sum_{jy}J_{ij}^{xy}F^{y}*s_{j}^{y}\left(t\right) \end{array}$$ (3)

where $J_{ij}^{xy}$ is the synaptic weight to neuron *i* in population *x* from neuron *j* in population *y*, *F*^*y*^ is the synaptic filter of neurons from population *y*, and $s_{j}^{y}\left(t\right)$ is an impulse train representing the spikes of neuron *j* from population *y*. The synaptic filter of excitatory and inhibitory neurons was modeled by a difference of exponentials via

$$\begin{array}{c} F^{y}\left(t\right)=\frac{1}{\tau_{2}-\tau_{1}}\left(e^{-t/\tau_{2}}-e^{-t/\tau_{1}}\right) \end{array}$$ (4)

where *τ*_2_ is 3 ms and 2 ms for excitatory and inhibitory neurons, respectively, *τ*_1_ is 1 ms for both neuron types, and *t* only takes positive values.

In the clustered network, same-cluster excitatory synaptic weights were set to $J_{IN}^{EE}=0.0456$, out-of-cluster weights were set to $J_{OUT}^{EE}=0.024$, and the rest of the weights were set to *J*^*EI*^ = −0.045, *J*^*IE*^ = 0.014, and *J*^*II*^ = −0.057. In the non-clustered network $J^{EE}=J_{OUT}^{EE}=0.024$, and the remaining synaptic weights were the same as in the clustered network.

### Factor analysis

In this study, population activity structure was characterized using factor analysis (FA) [27, 28, 36, 66, 67]. Unlike principal component analysis (PCA), FA partitions the spike count variance of each neuron into shared and independent components. This makes it possible for us to assess the shared population activity structure (i.e., the shared component), which can be obscured by Poisson-like spiking variability (i.e., the independent component). For this reason, FA is more appropriate than PCA for analyzing single-trial spike counts [37]. FA is defined as:

$$\begin{array}{c} x∼N(\mu ,LL^{T}+\Psi ) \end{array}$$ (5)

where $x\in R^{n\times 1}$ is a vector of spike counts across the *n* simultaneously-recorded neurons, $\mu \in R^{n\times 1}$ is a vector of mean spike counts, $L\in R^{n\times m}$ is the loading matrix relating *m* latent variables to the neural activity, and $\Psi \in R^{n\times n}$ is a diagonal matrix of independent variances for each neuron. The model parameters *μ*, *L*, and Ψ were estimated using the expectation maximization (EM) algorithm [68].

As shown in Fig 2A, FA separates spike count variance into a shared component *LL*^*T*^ and an independent component Ψ. The rank of *LL*^*T*^ is *m*, the number of latent dimensions needed to explain the shared population activity structure. To determine *m*, FA is applied to spike counts for various candidate values of *m*, and then we select the *m* that maximizes the cross-validated data likelihood using four folds.

In this study, we used two key metrics to summarize population activity structure: shared dimensionality (*d*_*shared*_) and percent shared variance. First, we measured the number of dimensions in the shared covariance as a metric for the complexity of the population activity. We followed a two step procedure to obtain this metric. We first found the *m* that maximized the cross-validated data likelihood, as is standard practice. We then defined *d*_*shared*_ as the number of dimensions that were needed to explain 95% of the shared variance based on the eigenvalues of *LL*^*T*^ (Fig 2B). We did this for the following reason. In simulations, we found that, when training data were abundant, there was not a strong effect of overfitting and the cross-validated data likelihood curve saturated at large dimensionalities. As a result, the peak data-likelihood appeared at widely varying dimensionalities along the flat portion of the curve, leading to variability in the value of *m* from one run to the next. In contrast, we found that defining *d*_*shared*_ as described above provided a more reliable estimate of dimensionality across analyses, even if it underestimates the true dimensionality.

Second, we measured the amount of each neuron’s variance that was shared with at least one other neuron in the sampled population (Fig 2C). Mathematically, percent shared variance for the *k*^*th*^ neuron was computed as:

$$\begin{array}{c} \text{Percent shared variance for neuron }k=\frac{L_{k}L_{k}^{T}}{L_{k}L_{k}^{T}+\Psi_{k}} \end{array}$$ (6)

where *L*_*k*_ is the *k*^*th*^ row of *L* and Ψ_*k*_ is the independent variance for the *k*^*th*^ neuron. In Figs 3, 5 and 6, we report averages over all neurons of the same type in a given analysis. For Figs 2B and 4B, we computed a separate metric, the percent of overall shared variance explained by each mode. This was used to quantify the relative dominance of each mode for explaining shared variability. The percent of shared variance explained by the *i*^*th*^ mode was computed as:

$$\begin{array}{c} \text{Percent of shared variance explained by mode }i=\frac{\lambda_{i}}{\sum_{j=1}^{m}\lambda_{j}} \end{array}$$ (7)

where λ_*i*_ is the eigenvalue of *LL*^*T*^ corresponding to the *i*^*th*^ mode and *m* is the rank of *L*. Note that this metric does not take into account the independent variances. For Fig 4, FA was applied with the cross-validated shared dimensionality to all 10,000 trials. Throughout this work, we refer to a particular mode as “dominant” to another mode if it explains a larger percent of shared variance.

### Varying neuron and trial count

We investigated how *d*_*shared*_ and percent shared variance vary with neuron and trial counts for excitatory and inhibitory populations in spiking network models and V1 recordings. To do so, we sampled increasing numbers of neurons or trials from either the network simulations or V1 recordings. FA was then applied to the selected neurons and trials to obtain *d*_*shared*_ and percent shared variance.

In the analysis of model networks in Fig 3, to increase neuron count, we augmented the next smaller sample of neurons with additional randomly selected neurons. For example, we first randomly selected 10 neurons, computed *d*_*shared*_ and percent shared variance for this set, and then added 10 additional randomly-selected neurons to obtain the next sample of neurons. We repeated this procedure 25 times at each neuron count using 5 non-overlapping sets of neurons and 5 non-overlapping sets of trials, using 10,000 trials for each neuron count. We studied how *d*_*shared*_ and percent shared variance change with trial count by performing the same procedure as described above, except that trials were increased rather than neurons.

For the V1 recording analysis in Fig 6A, we limited our analysis to 23 neurons of each type, since this was the minimum number of neurons of a particular type across the four arrays. For each neuron count, we took as many non-overlapping samples of neurons for that neuron count as possible, and applied FA to compute *d*_*shared*_ and percent shared variance. We applied the same sampling procedure to analyze how *d*_*shared*_ and percent shared variance change with increasing trial count, while limited to 1,200 trials. We repeated this procedure for each of the four arrays.

### Mixed neuron type sampling

Since realistic population samplings include a mixture of both excitatory and inhibitory neurons, we analyzed how the ratio of excitatory to inhibitory neurons sampled affects population activity structure. To do assess this, we fit FA to spiking network activity samplings of 100 neurons to compute *d*_*shared*_ and percent shared variance for ratios of 0:100, 10:90, 20:80, and so on until 100:0 excitatory to inhibitory neurons were sampled. Starting with the ratio of 0:100 excitatory to inhibitory neurons, we replaced 10 of the 100 inhibitory neurons with 10 excitatory neurons for a ratio of 10:90 excitatory to inhibitory neurons. Then, we replaced another 10 inhibitory neurons with 10 excitatory neurons (while keeping the 10 excitatory neurons just added to the sampling) for a ratio of 20:80 excitatory to inhibitory neurons sampled. Excitatory neurons replaced inhibitory neurons in increments of 10, until the entire sampling comprised zero inhibitory neurons and 100 excitatory neurons. We repeated this analysis 25 times for five non-overlapping sets of 100 excitatory and 100 inhibitory neurons and five non-overlapping sets of trials.

### Neural recordings

A detailed description of the recording methodology can be found in [40, 69]. Briefly, neurons were recorded in the primary visual cortex of three anesthetized macaque monkeys. Anesthesia was maintained for 5-7 days with sufentanil citrate (6-18 μg/kg/hr) through continuous intravenous infusion, and eye movements were reduced through continuous intravenous infusion of vecuronium bromide (100-150 μg/kg/hr).

Multi-electrode arrays were implanted in four primary visual cortex hemispheres of three anesthetized macaque monkeys, and neural activity was recorded for 20-30 minutes during presentation of a gray computer screen. Spiking waveform snippets from the recordings were sorted off-line using a mixture-decomposition method [70]. This automatic sorting was refined manually, taking into account waveform shape and inter-spike interval distributions. For our analysis, we only used neurons which had average firing rate greater than one spike per second, and signal-to-noise ratio (SNR), computed as the average waveform amplitude divided by the standard deviation across waveforms, greater than 1.33. Median SNR values were 2.96, 2.75, 2.50, and 2.31 for each of the four arrays. Spiking activity was divided into one second epochs, which we refer to as “trials” throughout this work. An analysis of a superset of these data can be found in [40].

Animal protocols were approved by the institutional animal care and use committees of New York University and Albert Einstein College of Medicine of Yeshiva University. Monkeys were fed nutrient-rich biscuits and frequently (1-2 times per day) given supplemental treats (e.g. fresh and dried fruit, nuts, etc). Enrichment activities included foraging for treats, music, movies, human interaction, and standard toys including mirrors. Animals were typically pair housed and, when this was not the case, animals were always housed in the same room as conspecifics, allowing frequent visual and auditory interactions. Professional veterinary staff and lab personnel monitored animal health and well-being on a daily basis.

### Neuron type classification

We classified the recorded neurons as putative excitatory or putative inhibitory neurons based on average waveform shape (S5 Fig). Compared to excitatory neurons, inhibitory neurons tend to have shorter latencies to peak depolarization [45–47], brief hyperpolarization durations [45, 46], and rapid peak rates of repolarization [45]. Neurons with any of these three properties are considered “narrow-spiking” (or fast-spiking), while neurons with slower action potential dynamics are considered “broad-spiking” (or regular-spiking). To classify neurons from our extracellular recordings as broad- or narrow-spiking (putative excitatory and putative inhibitory, respectively), we computed the time of peak depolarization, duration of hyperpolarization, and rate of repolarization for the average waveform of each neuron. We measured these three waveform statistics for the 75% of the neurons that across all recording sessions with the greaf SNR, and fit a mixture of two Gaussians to the scatter of waveforms, where each waveform was represented by its waveform statistics in the three-dimensional space [14]. The average waveforms of four neurons exhibited large increases in normalized amplitude before spike depolarization making them difficult to parameterize, so they were not used to train the mixture of Gaussians classifier or in the analyses. The class with the smaller mean time of peak depolarization was designated as the narrow-spiking class, and the other as the broad-spiking class. We used the fitted mixture of Gaussian parameters to compute the likelihood of a neuron being a part of either class. To focus on neurons that had a high probability of belonging to either class, we analyzed only neurons with probability greater than 85% of belonging to either class. This resulted in the following distributions of classified neuron types for the four arrays: 24 broad-spiking and 39 narrow-spiking, 41 broad-spiking and 72 narrow-spiking, 23 broad-spiking and 60 narrow-spiking, and 47 broad-spiking and 25 narrow-spiking neurons. These samples contain a smaller proportion of putative excitatory neurons than the 75-80% excitatory pyramidal cells found in histochemical analysis [71, 72]. Possible reasons for this include differences between the physiological and anatomical sampling distributions of neurons, ambiguity in classification due to the presence of some broad-spiking inhibitory neurons, differences in laminar distributions between cell types, and randomness in neural sampling for each array implant. Previous studies have not explored the large-scale distribution of cell types using this classification method, and this will be an important area for future research. Using the classifications we determined for each array implant, we restricted our analysis to a randomly-chosen set of 23 neurons for each neuron type for each array.

## Supporting information

S1 Fig Timescale of neuronal correlations. Autocorrelation of excitatory and inhibitory neurons using a 10 ms spike count window. Curves were averaged across neurons and have a maximum of one at zero time lag. (A) Clustered network: autocorrelation of excitatory neurons (red), inhibitory neurons (blue), and excitatory neurons broken down by cluster (orange). (B) Non-clustered network: autocorrelation of excitatory (red) and inhibitory (blue) neurons. (C) V1 recordings: autocorrelation of broad-spiking (red) and narrow-spiking (blue) neurons.
(EPS)

S2 Fig Relationship of percent shared variance to pairwise spike count correlation. To study the relationship of the width of a zero-mean spike count correlation distribution to shared variance, we generated simulated spike counts with near-zero mean spike count correlation distributions of various widths using the method described in [44]. We began by drawing spike count correlations from a normal distribution with mean zero and a selected standard deviation. They were used to form a correlation matrix, where the correlation values were placed randomly in the upper half of the matrix and were copied to the lower half of the matrix to ensure symmetry. For the correlation distribution widths tested here, all of the generated correlation matrices were positive semi-definite. The spike count means and variances of the model neurons were matched to randomly-sampled inhibitory neurons in the non-clustered network. We then generated 100,000 spike counts for 100 model neurons. We computed the percent shared variance for simulated spike counts for various correlation distribution widths. The widths 0.011 and 0.025 correspond to the excitatory (red) and inhibitory (blue) populations, respectively, in the non-clustered network (cf. Fig 1D, EE and II), and are thus highlighted. Standard error bars are shown for five sets of 20,000 trials, where each set is an independent draw from the distribution of spike count correlations.
(EPS)

S3 Fig Excitatory and inhibitory population activity structure using 100 ms spike count windows. Same analysis as shown in Fig 3 using a 100 ms spike count window. The same neurons and trials of the clustered and non-clustered networks were used. Spike counts were taken in the first 100 ms of the original one second trial.
(EPS)

S4 Fig Mixed neuron type samplings using 100 ms spike count windows. Same analysis as shown in Fig 5 using a 100 ms spike count window. The same neurons and trials of the clustered and non-clustered networks were used. Spike counts were taken in the first 100 ms of the original one second trials.
(EPS)

S5 Fig Neuron type classification. (A) Normalized average waveforms of neurons with average firing rates greater than one spike per second. Each waveform corresponds to one neuron and is colored by the probability that it belongs to the broad-spiking class (toward red) or the narrow-spiking class (toward blue). (B) Posterior probability of neurons belonging to either class. Neurons are ordered along the horizontal axis based on their relative probability of belonging to the broad-spiking class (red) and narrow-spiking class (blue). Dashed vertical lines indicate 85% probability thresholds used for determining neurons that clearly belong to each class. (C) Waveform shape averaged across all broad-spiking neurons (red) and all narrow-spiking neurons (blue) that passed the 85% probability threshold.
(EPS)

S6 Fig Modes of shared activity for V1 recordings. (A) Modes for broad-spiking neurons. The columns of the heatmap represent the eigenvectors of the shared covariance matrix, ordered by the amount of shared variance explained. (B) Same conventions as A for narrow-spiking neurons. (C) Percent of total shared variance of broad-spiking (red) and narrow-spiking (blue) neurons explained by each mode.
(EPS)
